# 4,6-Bis(diphenyl­phosphan­yl)dibenzo[*b*,*d*]furan

**DOI:** 10.1107/S1600536811047787

**Published:** 2011-11-16

**Authors:** Thashree Marimuthu, Holger B. Friedrich, Muhammad D. Bala

**Affiliations:** a School of Chemistry, University of KwaZulu-Natal, Westville Campus, Private Bag X54001, Durban 4000, South Africa

## Abstract

The asymmetric unit of the title compound, C_36_H_26_OP_2_, comprises two mol­ecules which have slightly different conformations of the phenyl ring substituents. In both mol­ecules, the dibenzofuran unit is close to being planar, with dihedral angles of 3.20 (3) and 1.86 (2)° for the two mol­ecules. Its planarity affects the intra­molecular distances between P atoms, with P⋯P distances of 5.574 (2) and 5.485 (2) Å for the two mol­ecules.

## Related literature

For related syntheses, see: Kranenburg *et al.* (1995 ▶); Hillebrand *et al.* (1995 ▶). For related structures, see: Vogl *et al.* (1996 ▶); Marimuthu *et al.* (2008*a* ▶,*b* ▶). For structures containing the dibenzofuran unit, see: Dideberg *et al.* (1972 ▶); Banerjee (1973 ▶); Reppart *et al.*, (1984 ▶).
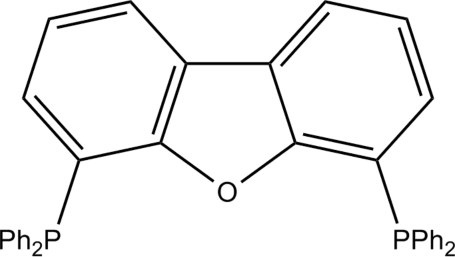


## Experimental

### 

#### Crystal data

C_36_H_26_OP_2_
*M* *_r_* = 536.51
Triclinic, 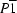

*a* = 11.7463 (2) Å
*b* = 14.7238 (3) Å
*c* = 16.7140 (4) Å
α = 96.821 (2)°
β = 96.351 (5)°
γ = 95.240 (2)°
*V* = 2836.58 (10) Å^3^
*Z* = 4
Mo *K*α radiation
μ = 0.18 mm^−1^
*T* = 173 K
0.40 × 0.22 × 0.18 mm

#### Data collection

Bruker APEXII CCD diffractometer
28406 measured reflections
12364 independent reflections
8323 reflections with *I* > 2σ(*I*)
*R*_int_ = 0.052

#### Refinement

*R*[*F* ^2^ > 2σ(*F* ^2^)] = 0.041
*wR*(*F* ^2^) = 0.100
*S* = 0.90
12363 reflections
703 parameters
H-atom parameters constrained
Δρ_max_ = 0.29 e Å^−3^
Δρ_min_ = −0.30 e Å^−3^

### 

Data collection: *APEX2* (Bruker, 2005 ▶); cell refinement: *SAINT-Plus* (Bruker, 2005 ▶); data reduction: *SAINT-Plus*; program(s) used to solve structure: *SHELXS97* (Sheldrick, 2008 ▶); program(s) used to refine structure: *SHELXL97* (Sheldrick, 2008 ▶); molecular graphics: *SHELXTL* (Sheldrick, 2008 ▶); software used to prepare material for publication: *SHELXTL*.

## Supplementary Material

Crystal structure: contains datablock(s) global, I. DOI: 10.1107/S1600536811047787/kp2367sup1.cif

Structure factors: contains datablock(s) I. DOI: 10.1107/S1600536811047787/kp2367Isup2.hkl

Supplementary material file. DOI: 10.1107/S1600536811047787/kp2367Isup3.cml

Additional supplementary materials:  crystallographic information; 3D view; checkCIF report

## Figures and Tables

**Fig. 1.**
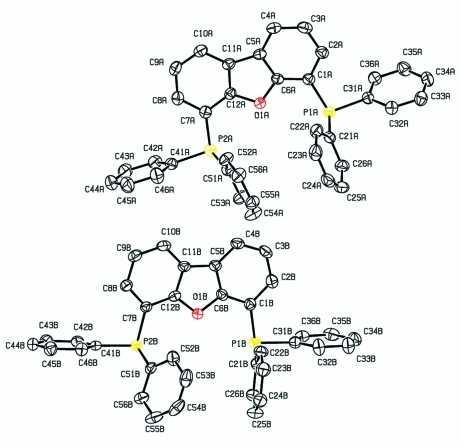
ORTEP diagram of compound (I). Displacement ellipsoids are represented at the 50% probability level. Hydrogen atoms omitted for clarity.

## supplementary crystallographic information

### Comment

The crystal structure of (I)comprises the two crystallographically independent conformers of the title compound (I)(Fig. 1). The dibenzofuran or aromatic ether backbone has been selectively functionalised with diphosphane donors. The diphosphane ligand has been used in Rh hydroformylation catalysis (Kranenburg *et al.*, 1995). In a study towards the isolation of stable coordination complexes, the complex of Co with a ligand (I) was published (Vogl *et al.* 1996); herein we report a different conformation of the ligand to the reported one. The individual five and six membered rings of dibenzofuran moiety in (I) are planar, but not coplanar; dihedral angles of 1.32° & 1.88° (molecule A) and 1.52° & 0.42° (molecule B) are observed. The planarity of the dibenzofuran moiety has been a subject of interest reporting the dihedral angles of 1.12° (Dideberg *et al.*, 1972), 1.1° (Banerjee, 1973) and 1.5° (Reppart *et al.*, 1984). However, (Vogl *et al.* 1996) have reported a larger dihedral angle of 2.45° and 2.51° which is attributed to the difference in the disposition of the phenyl rings as compared to previous reports and the title compound.

In a chelating diphosphane compound, the magnitude of the intramolecular separation distances between P atoms has a big impact on the stability of its coordination compounds. In the title compound, the rigid backbone pushes the diphosphane pincers apart resulting in relatively large intramolecular separation distances between the P atoms of 5.574 (2) and 5.485 (2) for molecules A and B, respectively. This value is slightly smaller than the 5.741 (1) reported by (Vogl *et al.* 1996), but notably larger than in xantphos, [4.025 (2) (Kranenburg *et al.*, 1995) and 4.046 Å (Hillebrand *et al.* 1995)] and nixantphos, 4.255 (2) Å (Marimuthu *et al.* 2008*a*).

### Experimental

The preparation of (I) was adapted from literature (Kranenburg *et al.*, 1995). A solution of dibenzofuran (1.5 g, 8.9 mmol) in 60 mL of dry ether was cooled to 208 K, using a dry ice/acetone bath. Thereafter, tetramethylethylenediamine (3.8 mL, 25 mmol) was added and the solution stirred. Under a positive pressure of argon, *n*BuLi (15.6 mL, 25 mmol) was added dropwise and the reaction left to stir for a further 16 h. Thereafter, the stirred reaction mixture was cooled to 208 K and chlorodiphenylphosphane (4.6 mL, 25 mmol) in hexane (13 mL) added dropwise. Then, the reaction was left to stir overnight. A (1/1) mixture of dichloromethane (DCM)/water (25 mL) was added. After allowing sufficient time to stir, the organic layer was removed, and the aqueous layer extracted with DCM (2 *x* 15 mL). The combined organic fractions were dried over anhydrous MgSO_4_, filtered, and the solvent evacuated to dryness. The resulting oil was washed with hexane and recrystalised from DCM/EtOH (1:1) to afford colourless crystals of (I) suitable for X-ray analysis. [yield: 3.4 g, 71%; m.p. 485 K]. Spectroscopic analysis: ^1^H NMR (400 MHz, CDCl_3_, δ, p.p.m.): 7.91 (dd, *J* = 7.7, 1.2 Hz, 2H), 7.44 – 7.09 (m, P(C_6_H_5_)_2_ and H xanthene, 22H), 7.03 (td, *J*(H,H) = 7.4, *J*(P,H) 1.2 Hz, 2H); ^13^C NMR (101 MHz, CDCl_3_, δ, p.p.m.): 157.9 (d, *J*(P,*C*) = 13.7 Hz, CO), 135.8 (d, *J*(P,*C*) = 10.7 Hz, phenyl C-*ipso*, PC), 133.8 (d, *J*(P,*C*) = 20.3 Hz, CH), 132.0 (d, *J* = 8.6 Hz, CH), 128.7 (s, CH), 128.3 (d, *J* (P,*C*) = 7.1 Hz, CH), 123.5 (bs, C), 123.1 (d, *J*(P,*C*) = 3.0 Hz, CH), 121.2 (d, *J* (P,*C*) = 19.5 Hz, C), 121.5 (CH); ^31^P NMR (243 MHz, CDCl_3_δ, p.p.m.): -16.9; IR (neat, ν_max_, cm^-1^): 3046 (*w*), 3006 (*w*), 1476 (*m*), 1433 (*s*), 1408 (*s*), 1387 (*m*), 1173 (*s*), 772 (*m*), 739 (*s*), 691 (*s*); HR—MS (ESI) (m/*z*): 537.1531 [*M* + H]^+^ calcd. for C_36_H_27_OP_2_, 537.1532.

### Refinement

All H-atoms were refined using a riding model, with C—H = 0.95 Å and *U*_iso_(H) = 1.2*U*_eq_(C).

### Crystal data

**Table:**

C_36_H_26_OP_2_	*Z* = 4
*M**_r_* = 536.51	*F*(000) = 1120
Triclinic, *P*1	*D*_x_ = 1.256 Mg m^−^^3^
Hall symbol: -P 1	Melting point: 485 K
*a* = 11.7463 (2) Å	Mo *K*α radiation, λ = 0.71073 Å
*b* = 14.7238 (3) Å	Cell parameters from 7048 reflections
*c* = 16.7140 (4) Å	θ = 2.3–26.9°
α = 96.821 (2)°	µ = 0.18 mm^−^^1^
β = 96.351 (5)°	*T* = 173 K
γ = 95.240 (2)°	Prism, colourless
*V* = 2836.58 (10) Å^3^	0.40 × 0.22 × 0.18 mm

### Data collection

**Table:**

Bruker APEXII CCD diffractometer	8323 reflections with *I* > 2σ(*I*)
Radiation source: fine-focus sealed tube	*R*_int_ = 0.052
graphite	θ_max_ = 27.0°, θ_min_ = 1.2°
φ and ω scans	*h* = −15→14
28406 measured reflections	*k* = −14→18
12364 independent reflections	*l* = −21→19

### Refinement

**Table:**

Refinement on *F*^2^	Primary atom site location: structure-invariant direct methods
Least-squares matrix: full	Secondary atom site location: difference Fourier map
*R*[*F*^2^ > 2σ(*F*^2^)] = 0.041	Hydrogen site location: inferred from neighbouring sites
*wR*(*F*^2^) = 0.100	H-atom parameters constrained
*S* = 0.90	*w* = 1/[σ^2^(*F*_o_^2^) + (0.0468*P*)^2^] where *P* = (*F*_o_^2^ + 2*F*_c_^2^)/3
12363 reflections	(Δ/σ)_max_ = 0.002
703 parameters	Δρ_max_ = 0.29 e Å^−^^3^
0 restraints	Δρ_min_ = −0.30 e Å^−^^3^

### Special details

**Table:**

Geometry. All e.s.d.'s (except the e.s.d. in the dihedral angle between two l.s. planes) are estimated using the full covariance matrix. The cell e.s.d.'s are taken into account individually in the estimation of e.s.d.'s in distances, angles and torsion angles; correlations between e.s.d.'s in cell parameters are only used when they are defined by crystal symmetry. An approximate (isotropic) treatment of cell e.s.d.'s is used for estimating e.s.d.'s involving l.s. planes.
Refinement. Refinement of *F*^2^ against ALL reflections. The weighted *R*-factor *wR* and goodness of fit *S* are based on *F*^2^, conventional *R*-factors *R* are based on *F*, with *F* set to zero for negative *F*^2^. The threshold expression of *F*^2^ > σ(*F*^2^) is used only for calculating *R*-factors(gt) *etc*. and is not relevant to the choice of reflections for refinement. *R*-factors based on *F*^2^ are statistically about twice as large as those based on *F*, and *R*- factors based on ALL data will be even larger.One reflection was an outlier.

### Fractional atomic coordinates and isotropic or equivalent isotropic displacement parameters (Å^2^)

**Table:**

	*x*	*y*	*z*	*U*_iso_*/*U*_eq_	
C1A	0.37941 (15)	0.82094 (11)	0.53677 (10)	0.0274 (4)	
C2A	0.31908 (16)	0.88254 (12)	0.49585 (11)	0.0332 (4)	
H2A	0.3066	0.8730	0.4382	0.040*	
C3A	0.27640 (17)	0.95794 (12)	0.53724 (12)	0.0370 (5)	
H3A	0.2357	0.9981	0.5071	0.044*	
C4A	0.29204 (16)	0.97513 (12)	0.62065 (12)	0.0335 (4)	
H4A	0.2625	1.0264	0.6482	0.040*	
C5A	0.35234 (16)	0.91537 (11)	0.66360 (11)	0.0285 (4)	
C6A	0.39397 (15)	0.84093 (11)	0.62080 (11)	0.0268 (4)	
C7A	0.48344 (15)	0.79506 (11)	0.82089 (11)	0.0282 (4)	
C8A	0.46446 (16)	0.84741 (12)	0.89246 (11)	0.0333 (4)	
H8A	0.4901	0.8277	0.9429	0.040*	
C9A	0.40934 (17)	0.92762 (13)	0.89305 (12)	0.0373 (5)	
H9A	0.4002	0.9614	0.9436	0.045*	
C10A	0.36791 (17)	0.95880 (12)	0.82206 (11)	0.0340 (5)	
H10A	0.3293	1.0128	0.8228	0.041*	
C11A	0.38466 (15)	0.90833 (11)	0.74890 (11)	0.0284 (4)	
C12A	0.44181 (15)	0.82992 (11)	0.75056 (10)	0.0270 (4)	
C21A	0.33887 (16)	0.62567 (11)	0.49008 (10)	0.0291 (4)	
C22A	0.23537 (17)	0.63032 (13)	0.52293 (11)	0.0366 (5)	
H22A	0.2125	0.6885	0.5417	0.044*	
C23A	0.16524 (19)	0.55069 (14)	0.52854 (12)	0.0440 (5)	
H23A	0.0955	0.5547	0.5520	0.053*	
C24A	0.1962 (2)	0.46631 (13)	0.50037 (12)	0.0431 (5)	
H24A	0.1477	0.4121	0.5041	0.052*	
C25A	0.2978 (2)	0.45991 (13)	0.46652 (12)	0.0438 (5)	
H25A	0.3189	0.4015	0.4463	0.053*	
C26A	0.36865 (18)	0.53926 (12)	0.46226 (12)	0.0378 (5)	
H26A	0.4391	0.5346	0.4399	0.045*	
C31A	0.42922 (15)	0.74431 (11)	0.38134 (10)	0.0278 (4)	
C32A	0.35837 (16)	0.68820 (12)	0.31898 (11)	0.0317 (4)	
H32A	0.3069	0.6399	0.3316	0.038*	
C33A	0.36212 (17)	0.70197 (13)	0.23882 (12)	0.0375 (5)	
H33A	0.3141	0.6624	0.1970	0.045*	
C34A	0.43455 (17)	0.77238 (13)	0.21902 (12)	0.0386 (5)	
H34A	0.4369	0.7814	0.1639	0.046*	
C35A	0.50414 (18)	0.83009 (13)	0.28039 (12)	0.0404 (5)	
H35A	0.5533	0.8797	0.2674	0.048*	
C36A	0.50225 (17)	0.81565 (12)	0.36026 (11)	0.0351 (5)	
H36A	0.5514	0.8549	0.4017	0.042*	
C41A	0.58347 (16)	0.67629 (11)	0.92241 (11)	0.0302 (4)	
C42A	0.68812 (17)	0.71181 (12)	0.96745 (12)	0.0360 (5)	
H42A	0.7459	0.7422	0.9416	0.043*	
C43A	0.7095 (2)	0.70373 (14)	1.04910 (13)	0.0472 (6)	
H43A	0.7812	0.7291	1.0789	0.057*	
C44A	0.6276 (2)	0.65914 (14)	1.08731 (13)	0.0518 (6)	
H44A	0.6424	0.6536	1.1435	0.062*	
C45A	0.5234 (2)	0.62240 (14)	1.04374 (13)	0.0530 (6)	
H45A	0.4668	0.5912	1.0699	0.064*	
C46A	0.50146 (19)	0.63084 (13)	0.96218 (12)	0.0442 (5)	
H46A	0.4295	0.6053	0.9328	0.053*	
C51A	0.44897 (15)	0.60216 (11)	0.77260 (10)	0.0265 (4)	
C52A	0.33230 (16)	0.61084 (12)	0.77160 (11)	0.0357 (5)	
H52A	0.3082	0.6676	0.7932	0.043*	
C53A	0.25030 (17)	0.53820 (12)	0.73967 (11)	0.0376 (5)	
H53A	0.1707	0.5454	0.7392	0.045*	
C54A	0.28428 (18)	0.45581 (13)	0.70864 (11)	0.0378 (5)	
H54A	0.2282	0.4059	0.6867	0.045*	
C55A	0.39956 (19)	0.44551 (13)	0.70934 (13)	0.0466 (6)	
H55A	0.4230	0.3883	0.6884	0.056*	
C56A	0.48183 (17)	0.51863 (13)	0.74064 (12)	0.0398 (5)	
H56A	0.5613	0.5113	0.7401	0.048*	
O1A	0.44942 (10)	0.78761 (7)	0.67250 (7)	0.0282 (3)	
P1A	0.44520 (4)	0.72421 (3)	0.48805 (3)	0.02816 (12)	
P2A	0.56344 (4)	0.69353 (3)	0.81530 (3)	0.02891 (12)	
C1B	0.00064 (16)	0.29822 (12)	0.74027 (11)	0.0290 (4)	
C2B	−0.01889 (16)	0.35240 (12)	0.67774 (11)	0.0338 (4)	
H2B	0.0026	0.3324	0.6259	0.041*	
C3B	−0.06894 (17)	0.43487 (12)	0.68878 (11)	0.0355 (5)	
H3B	−0.0795	0.4697	0.6447	0.043*	
C4B	−0.10324 (16)	0.46657 (12)	0.76244 (11)	0.0326 (4)	
H4B	−0.1380	0.5223	0.7694	0.039*	
C5B	−0.08556 (15)	0.41471 (11)	0.82632 (11)	0.0280 (4)	
C6B	−0.03372 (15)	0.33400 (11)	0.81348 (10)	0.0269 (4)	
C7B	−0.07776 (15)	0.32607 (11)	1.01918 (10)	0.0257 (4)	
C8B	−0.12956 (16)	0.39021 (11)	1.06783 (11)	0.0307 (4)	
H8B	−0.1366	0.3810	1.1225	0.037*	
C9B	−0.17122 (17)	0.46730 (12)	1.03859 (12)	0.0354 (5)	
H9B	−0.2063	0.5089	1.0737	0.043*	
C10B	−0.16281 (16)	0.48458 (12)	0.96022 (11)	0.0335 (4)	
H10B	−0.1914	0.5373	0.9410	0.040*	
C11B	−0.11105 (16)	0.42244 (11)	0.90970 (11)	0.0277 (4)	
C12B	−0.07107 (15)	0.34590 (11)	0.94068 (10)	0.0258 (4)	
C21B	−0.04604 (17)	0.10425 (12)	0.73400 (10)	0.0297 (4)	
C22B	−0.16215 (17)	0.11507 (13)	0.71759 (11)	0.0369 (5)	
H22B	−0.1842	0.1739	0.7086	0.044*	
C23B	−0.24634 (19)	0.04216 (13)	0.71404 (12)	0.0423 (5)	
H23B	−0.3252	0.0509	0.7021	0.051*	
C24B	−0.2155 (2)	−0.04334 (13)	0.72784 (12)	0.0450 (6)	
H24B	−0.2730	−0.0936	0.7256	0.054*	
C25B	−0.1012 (2)	−0.05521 (14)	0.74479 (13)	0.0513 (6)	
H25B	−0.0797	−0.1140	0.7543	0.062*	
C26B	−0.01721 (19)	0.01758 (13)	0.74821 (12)	0.0422 (5)	
H26B	0.0615	0.0083	0.7605	0.051*	
C31B	0.09720 (16)	0.17990 (12)	0.62744 (11)	0.0307 (4)	
C32B	0.01672 (18)	0.13673 (13)	0.56366 (11)	0.0403 (5)	
H32B	−0.0558	0.1104	0.5747	0.048*	
C33B	0.04143 (19)	0.13184 (14)	0.48452 (12)	0.0455 (5)	
H33B	−0.0141	0.1019	0.4416	0.055*	
C34B	0.14588 (19)	0.17008 (13)	0.46724 (12)	0.0420 (5)	
H34B	0.1620	0.1674	0.4126	0.050*	
C35B	0.22697 (18)	0.21236 (13)	0.52976 (13)	0.0402 (5)	
H35B	0.2993	0.2386	0.5182	0.048*	
C36B	0.20306 (17)	0.21651 (12)	0.60921 (12)	0.0348 (5)	
H36B	0.2599	0.2448	0.6520	0.042*	
C41B	−0.01766 (15)	0.24706 (10)	1.16214 (10)	0.0247 (4)	
C42B	0.08149 (16)	0.28867 (11)	1.21114 (11)	0.0301 (4)	
H42B	0.1489	0.3044	1.1870	0.036*	
C43B	0.08319 (17)	0.30748 (12)	1.29468 (11)	0.0343 (5)	
H43B	0.1510	0.3368	1.3273	0.041*	
C44B	−0.01381 (18)	0.28352 (12)	1.33004 (11)	0.0350 (5)	
H44B	−0.0130	0.2966	1.3872	0.042*	
C45B	−0.11229 (17)	0.24048 (12)	1.28261 (11)	0.0363 (5)	
H45B	−0.1787	0.2233	1.3073	0.044*	
C46B	−0.11418 (16)	0.22246 (12)	1.19921 (11)	0.0315 (4)	
H46B	−0.1822	0.1930	1.1670	0.038*	
C51B	−0.11728 (16)	0.13006 (11)	1.01669 (10)	0.0279 (4)	
C52B	−0.20495 (17)	0.13010 (13)	0.95380 (11)	0.0387 (5)	
H52B	−0.2184	0.1857	0.9325	0.046*	
C53B	−0.27329 (19)	0.04889 (15)	0.92185 (12)	0.0477 (6)	
H53B	−0.3328	0.0493	0.8785	0.057*	
C54B	−0.25549 (19)	−0.03169 (14)	0.95239 (14)	0.0479 (6)	
H54B	−0.3021	−0.0870	0.9300	0.058*	
C55B	−0.16983 (19)	−0.03237 (13)	1.01565 (14)	0.0454 (6)	
H55B	−0.1582	−0.0879	1.0376	0.055*	
C56B	−0.10075 (17)	0.04794 (12)	1.04714 (12)	0.0361 (5)	
H56B	−0.0411	0.0468	1.0902	0.043*	
O1B	−0.02303 (10)	0.29065 (7)	0.88294 (7)	0.0272 (3)	
P1B	0.07320 (4)	0.19318 (3)	0.73453 (3)	0.03273 (12)	
P2B	−0.00896 (4)	0.22826 (3)	1.05283 (3)	0.02579 (11)	

### Atomic displacement parameters (Å^2^)

**Table:**

	*U*^11^	*U*^22^	*U*^33^	*U*^12^	*U*^13^	*U*^23^
C1A	0.0267 (10)	0.0243 (9)	0.0301 (10)	−0.0013 (7)	0.0008 (8)	0.0037 (7)
C2A	0.0340 (11)	0.0309 (10)	0.0334 (11)	0.0027 (8)	−0.0024 (9)	0.0052 (8)
C3A	0.0363 (12)	0.0289 (10)	0.0453 (13)	0.0059 (8)	−0.0043 (9)	0.0089 (9)
C4A	0.0326 (11)	0.0233 (9)	0.0434 (12)	0.0034 (8)	0.0029 (9)	0.0017 (8)
C5A	0.0284 (10)	0.0216 (9)	0.0348 (11)	−0.0011 (7)	0.0040 (8)	0.0027 (7)
C6A	0.0253 (10)	0.0239 (9)	0.0309 (10)	−0.0003 (7)	0.0018 (8)	0.0059 (7)
C7A	0.0259 (10)	0.0286 (10)	0.0295 (10)	−0.0011 (7)	0.0046 (8)	0.0036 (8)
C8A	0.0326 (11)	0.0368 (11)	0.0298 (11)	0.0001 (8)	0.0044 (8)	0.0038 (8)
C9A	0.0419 (13)	0.0374 (11)	0.0309 (11)	0.0025 (9)	0.0088 (9)	−0.0047 (9)
C10A	0.0365 (12)	0.0269 (10)	0.0388 (12)	0.0027 (8)	0.0095 (9)	0.0007 (8)
C11A	0.0279 (10)	0.0232 (9)	0.0333 (10)	−0.0016 (7)	0.0055 (8)	0.0017 (7)
C12A	0.0275 (10)	0.0257 (9)	0.0267 (10)	−0.0006 (7)	0.0051 (8)	−0.0005 (7)
C21A	0.0320 (11)	0.0275 (10)	0.0269 (10)	0.0021 (8)	−0.0018 (8)	0.0057 (7)
C22A	0.0424 (13)	0.0325 (11)	0.0344 (11)	−0.0005 (9)	0.0085 (9)	0.0016 (8)
C23A	0.0474 (14)	0.0473 (13)	0.0355 (12)	−0.0084 (10)	0.0100 (10)	0.0036 (9)
C24A	0.0562 (15)	0.0332 (11)	0.0363 (12)	−0.0086 (10)	−0.0071 (10)	0.0116 (9)
C25A	0.0564 (15)	0.0279 (11)	0.0440 (13)	0.0066 (10)	−0.0086 (11)	0.0044 (9)
C26A	0.0389 (12)	0.0333 (11)	0.0412 (12)	0.0096 (9)	−0.0013 (9)	0.0063 (9)
C31A	0.0250 (10)	0.0304 (10)	0.0287 (10)	0.0052 (7)	0.0037 (8)	0.0047 (8)
C32A	0.0262 (10)	0.0353 (10)	0.0325 (11)	−0.0001 (8)	0.0026 (8)	0.0038 (8)
C33A	0.0339 (12)	0.0423 (12)	0.0336 (11)	0.0019 (9)	−0.0002 (9)	−0.0007 (9)
C34A	0.0399 (13)	0.0481 (12)	0.0310 (11)	0.0102 (9)	0.0074 (9)	0.0104 (9)
C35A	0.0405 (13)	0.0405 (12)	0.0408 (12)	−0.0022 (9)	0.0059 (10)	0.0125 (9)
C36A	0.0340 (12)	0.0347 (11)	0.0352 (11)	−0.0010 (8)	0.0013 (9)	0.0046 (8)
C41A	0.0312 (11)	0.0276 (10)	0.0310 (10)	0.0043 (8)	0.0004 (8)	0.0035 (8)
C42A	0.0332 (12)	0.0358 (11)	0.0376 (12)	0.0073 (8)	−0.0012 (9)	0.0013 (9)
C43A	0.0475 (14)	0.0491 (13)	0.0408 (13)	0.0166 (10)	−0.0117 (11)	−0.0041 (10)
C44A	0.0743 (18)	0.0514 (14)	0.0307 (12)	0.0215 (12)	−0.0020 (12)	0.0063 (10)
C45A	0.0734 (18)	0.0481 (13)	0.0387 (13)	−0.0017 (12)	0.0127 (12)	0.0111 (10)
C46A	0.0456 (14)	0.0480 (13)	0.0358 (12)	−0.0082 (10)	0.0007 (10)	0.0069 (9)
C51A	0.0274 (10)	0.0290 (9)	0.0229 (9)	0.0044 (8)	0.0016 (8)	0.0031 (7)
C52A	0.0309 (11)	0.0328 (10)	0.0407 (12)	0.0070 (8)	0.0023 (9)	−0.0068 (8)
C53A	0.0281 (11)	0.0396 (11)	0.0421 (12)	0.0028 (8)	0.0005 (9)	−0.0032 (9)
C54A	0.0367 (12)	0.0330 (11)	0.0398 (12)	−0.0010 (9)	−0.0004 (9)	−0.0024 (9)
C55A	0.0458 (14)	0.0311 (11)	0.0597 (15)	0.0070 (9)	0.0067 (11)	−0.0094 (10)
C56A	0.0300 (11)	0.0385 (11)	0.0508 (13)	0.0094 (9)	0.0068 (9)	−0.0013 (9)
O1A	0.0334 (7)	0.0257 (6)	0.0256 (7)	0.0053 (5)	0.0030 (5)	0.0028 (5)
P1A	0.0277 (3)	0.0285 (3)	0.0279 (3)	0.00275 (19)	0.0020 (2)	0.00360 (19)
P2A	0.0252 (3)	0.0323 (3)	0.0292 (3)	0.0020 (2)	0.0033 (2)	0.0044 (2)
C1B	0.0288 (10)	0.0293 (10)	0.0274 (10)	−0.0002 (8)	0.0008 (8)	0.0030 (7)
C2B	0.0347 (12)	0.0373 (11)	0.0280 (10)	−0.0010 (9)	0.0013 (8)	0.0052 (8)
C3B	0.0409 (12)	0.0313 (10)	0.0342 (11)	−0.0014 (9)	−0.0019 (9)	0.0144 (8)
C4B	0.0360 (12)	0.0228 (9)	0.0378 (11)	−0.0007 (8)	−0.0006 (9)	0.0070 (8)
C5B	0.0292 (10)	0.0219 (9)	0.0313 (10)	−0.0022 (7)	−0.0003 (8)	0.0041 (7)
C6B	0.0282 (10)	0.0258 (9)	0.0258 (10)	−0.0018 (7)	−0.0007 (8)	0.0066 (7)
C7B	0.0282 (10)	0.0208 (9)	0.0278 (10)	0.0007 (7)	0.0045 (8)	0.0021 (7)
C8B	0.0343 (11)	0.0276 (10)	0.0304 (10)	0.0038 (8)	0.0061 (8)	0.0024 (8)
C9B	0.0390 (12)	0.0287 (10)	0.0392 (12)	0.0084 (8)	0.0093 (9)	−0.0007 (8)
C10B	0.0369 (12)	0.0226 (9)	0.0403 (12)	0.0053 (8)	0.0007 (9)	0.0042 (8)
C11B	0.0299 (10)	0.0202 (9)	0.0317 (10)	−0.0013 (7)	0.0007 (8)	0.0038 (7)
C12B	0.0251 (10)	0.0213 (9)	0.0297 (10)	0.0011 (7)	0.0030 (8)	0.0002 (7)
C21B	0.0398 (12)	0.0309 (10)	0.0210 (9)	0.0118 (8)	0.0053 (8)	0.0061 (7)
C22B	0.0424 (13)	0.0302 (10)	0.0420 (12)	0.0123 (9)	0.0090 (10)	0.0113 (9)
C23B	0.0431 (13)	0.0413 (12)	0.0443 (13)	0.0068 (10)	0.0103 (10)	0.0067 (9)
C24B	0.0648 (17)	0.0334 (11)	0.0374 (12)	0.0007 (10)	0.0123 (11)	0.0048 (9)
C25B	0.0769 (19)	0.0294 (11)	0.0506 (14)	0.0166 (11)	0.0066 (13)	0.0112 (10)
C26B	0.0516 (14)	0.0396 (12)	0.0384 (12)	0.0216 (10)	0.0027 (10)	0.0074 (9)
C31B	0.0325 (11)	0.0307 (10)	0.0298 (10)	0.0086 (8)	0.0030 (8)	0.0038 (8)
C32B	0.0354 (12)	0.0540 (13)	0.0304 (11)	−0.0028 (10)	0.0040 (9)	0.0069 (9)
C33B	0.0507 (15)	0.0538 (13)	0.0306 (12)	0.0023 (11)	0.0021 (10)	0.0044 (9)
C34B	0.0535 (15)	0.0456 (12)	0.0343 (12)	0.0189 (10)	0.0169 (10)	0.0141 (9)
C35B	0.0363 (12)	0.0365 (11)	0.0542 (14)	0.0099 (9)	0.0176 (10)	0.0158 (10)
C36B	0.0340 (12)	0.0281 (10)	0.0427 (12)	0.0065 (8)	0.0036 (9)	0.0045 (8)
C41B	0.0297 (10)	0.0182 (8)	0.0269 (10)	0.0054 (7)	0.0034 (8)	0.0030 (7)
C42B	0.0334 (11)	0.0240 (9)	0.0322 (10)	−0.0024 (8)	0.0037 (8)	0.0053 (7)
C43B	0.0405 (12)	0.0261 (10)	0.0328 (11)	−0.0017 (8)	−0.0049 (9)	0.0025 (8)
C44B	0.0481 (13)	0.0315 (10)	0.0254 (10)	0.0087 (9)	0.0023 (9)	0.0027 (8)
C45B	0.0355 (12)	0.0401 (11)	0.0349 (11)	0.0065 (9)	0.0098 (9)	0.0048 (9)
C46B	0.0279 (11)	0.0344 (10)	0.0313 (11)	0.0025 (8)	0.0027 (8)	0.0025 (8)
C51B	0.0312 (11)	0.0264 (9)	0.0260 (10)	0.0012 (8)	0.0101 (8)	−0.0013 (7)
C52B	0.0429 (13)	0.0396 (11)	0.0317 (11)	−0.0064 (9)	0.0039 (9)	0.0053 (8)
C53B	0.0458 (14)	0.0568 (14)	0.0339 (12)	−0.0146 (11)	0.0044 (10)	−0.0056 (10)
C54B	0.0477 (14)	0.0359 (12)	0.0550 (15)	−0.0118 (10)	0.0212 (12)	−0.0163 (10)
C55B	0.0470 (14)	0.0234 (10)	0.0676 (16)	0.0029 (9)	0.0217 (12)	−0.0004 (10)
C56B	0.0372 (12)	0.0291 (10)	0.0429 (12)	0.0054 (8)	0.0111 (9)	0.0012 (8)
O1B	0.0333 (7)	0.0243 (6)	0.0245 (7)	0.0057 (5)	0.0038 (5)	0.0034 (5)
P1B	0.0339 (3)	0.0384 (3)	0.0262 (3)	0.0103 (2)	0.0005 (2)	0.0032 (2)
P2B	0.0272 (3)	0.0238 (2)	0.0267 (3)	0.00263 (19)	0.0049 (2)	0.00286 (18)

### Geometric parameters (Å, °)

**Table:**

C1A—C6A	1.388 (2)	C1B—C6B	1.390 (2)
C1A—C2A	1.396 (2)	C1B—C2B	1.400 (2)
C1A—P1A	1.8312 (18)	C1B—P1B	1.8306 (19)
C2A—C3A	1.399 (3)	C2B—C3B	1.398 (3)
C2A—H2A	0.9500	C2B—H2B	0.9500
C3A—C4A	1.376 (3)	C3B—C4B	1.380 (3)
C3A—H3A	0.9500	C3B—H3B	0.9500
C4A—C5A	1.394 (3)	C4B—C5B	1.393 (2)
C4A—H4A	0.9500	C4B—H4B	0.9500
C5A—C6A	1.393 (2)	C5B—C6B	1.389 (2)
C5A—C11A	1.453 (2)	C5B—C11B	1.451 (2)
C6A—O1A	1.385 (2)	C6B—O1B	1.3890 (19)
C7A—C8A	1.395 (2)	C7B—C12B	1.387 (2)
C7A—C12A	1.395 (2)	C7B—C8B	1.398 (2)
C7A—P2A	1.8355 (19)	C7B—P2B	1.8296 (18)
C8A—C9A	1.397 (3)	C8B—C9B	1.395 (2)
C8A—H8A	0.9500	C8B—H8B	0.9500
C9A—C10A	1.378 (3)	C9B—C10B	1.376 (3)
C9A—H9A	0.9500	C9B—H9B	0.9500
C10A—C11A	1.396 (2)	C10B—C11B	1.397 (2)
C10A—H10A	0.9500	C10B—H10B	0.9500
C11A—C12A	1.389 (2)	C11B—C12B	1.393 (2)
C12A—O1A	1.3933 (19)	C12B—O1B	1.3891 (19)
C21A—C26A	1.391 (2)	C21B—C22B	1.389 (3)
C21A—C22A	1.392 (3)	C21B—C26B	1.390 (2)
C21A—P1A	1.8320 (17)	C21B—P1B	1.8264 (19)
C22A—C23A	1.389 (2)	C22B—C23B	1.383 (3)
C22A—H22A	0.9500	C22B—H22B	0.9500
C23A—C24A	1.371 (3)	C23B—C24B	1.379 (3)
C23A—H23A	0.9500	C23B—H23B	0.9500
C24A—C25A	1.382 (3)	C24B—C25B	1.373 (3)
C24A—H24A	0.9500	C24B—H24B	0.9500
C25A—C26A	1.385 (3)	C25B—C26B	1.380 (3)
C25A—H25A	0.9500	C25B—H25B	0.9500
C26A—H26A	0.9500	C26B—H26B	0.9500
C31A—C32A	1.393 (2)	C31B—C36B	1.389 (3)
C31A—C36A	1.398 (2)	C31B—C32B	1.393 (2)
C31A—P1A	1.8344 (18)	C31B—P1B	1.8331 (18)
C32A—C33A	1.383 (2)	C32B—C33B	1.380 (3)
C32A—H32A	0.9500	C32B—H32B	0.9500
C33A—C34A	1.375 (3)	C33B—C34B	1.377 (3)
C33A—H33A	0.9500	C33B—H33B	0.9500
C34A—C35A	1.387 (3)	C34B—C35B	1.380 (3)
C34A—H34A	0.9500	C34B—H34B	0.9500
C35A—C36A	1.379 (3)	C35B—C36B	1.383 (3)
C35A—H35A	0.9500	C35B—H35B	0.9500
C36A—H36A	0.9500	C36B—H36B	0.9500
C41A—C42A	1.391 (2)	C41B—C46B	1.391 (2)
C41A—C46A	1.395 (3)	C41B—C42B	1.393 (2)
C41A—P2A	1.8305 (19)	C41B—P2B	1.8308 (17)
C42A—C43A	1.381 (3)	C42B—C43B	1.388 (2)
C42A—H42A	0.9500	C42B—H42B	0.9500
C43A—C44A	1.373 (3)	C43B—C44B	1.377 (3)
C43A—H43A	0.9500	C43B—H43B	0.9500
C44A—C45A	1.381 (3)	C44B—C45B	1.383 (3)
C44A—H44A	0.9500	C44B—H44B	0.9500
C45A—C46A	1.382 (3)	C45B—C46B	1.385 (2)
C45A—H45A	0.9500	C45B—H45B	0.9500
C46A—H46A	0.9500	C46B—H46B	0.9500
C51A—C56A	1.387 (2)	C51B—C56B	1.388 (2)
C51A—C52A	1.386 (2)	C51B—C52B	1.388 (3)
C51A—P2A	1.8321 (17)	C51B—P2B	1.8333 (17)
C52A—C53A	1.385 (2)	C52B—C53B	1.392 (2)
C52A—H52A	0.9500	C52B—H52B	0.9500
C53A—C54A	1.373 (3)	C53B—C54B	1.370 (3)
C53A—H53A	0.9500	C53B—H53B	0.9500
C54A—C55A	1.375 (3)	C54B—C55B	1.378 (3)
C54A—H54A	0.9500	C54B—H54B	0.9500
C55A—C56A	1.390 (3)	C55B—C56B	1.384 (2)
C55A—H55A	0.9500	C55B—H55B	0.9500
C56A—H56A	0.9500	C56B—H56B	0.9500
			
C6A—C1A—C2A	114.83 (16)	C6B—C1B—C2B	114.00 (17)
C6A—C1A—P1A	119.91 (14)	C6B—C1B—P1B	119.76 (14)
C2A—C1A—P1A	125.14 (14)	C2B—C1B—P1B	126.11 (14)
C1A—C2A—C3A	121.96 (18)	C3B—C2B—C1B	122.40 (17)
C1A—C2A—H2A	119.0	C3B—C2B—H2B	118.8
C3A—C2A—H2A	119.0	C1B—C2B—H2B	118.8
C4A—C3A—C2A	121.47 (18)	C4B—C3B—C2B	121.25 (17)
C4A—C3A—H3A	119.3	C4B—C3B—H3B	119.4
C2A—C3A—H3A	119.3	C2B—C3B—H3B	119.4
C3A—C4A—C5A	118.23 (17)	C3B—C4B—C5B	118.25 (18)
C3A—C4A—H4A	120.9	C3B—C4B—H4B	120.9
C5A—C4A—H4A	120.9	C5B—C4B—H4B	120.9
C6A—C5A—C4A	119.06 (17)	C6B—C5B—C4B	118.87 (17)
C6A—C5A—C11A	105.72 (16)	C6B—C5B—C11B	106.04 (15)
C4A—C5A—C11A	135.21 (17)	C4B—C5B—C11B	135.08 (17)
O1A—C6A—C1A	123.88 (16)	C5B—C6B—O1B	111.51 (15)
O1A—C6A—C5A	111.64 (15)	C5B—C6B—C1B	125.21 (17)
C1A—C6A—C5A	124.45 (17)	O1B—C6B—C1B	123.25 (16)
C8A—C7A—C12A	113.82 (17)	C12B—C7B—C8B	114.56 (16)
C8A—C7A—P2A	125.20 (15)	C12B—C7B—P2B	119.20 (13)
C12A—C7A—P2A	120.86 (13)	C8B—C7B—P2B	125.98 (14)
C7A—C8A—C9A	122.72 (18)	C9B—C8B—C7B	122.00 (17)
C7A—C8A—H8A	118.6	C9B—C8B—H8B	119.0
C9A—C8A—H8A	118.6	C7B—C8B—H8B	119.0
C10A—C9A—C8A	121.52 (17)	C10B—C9B—C8B	121.65 (17)
C10A—C9A—H9A	119.2	C10B—C9B—H9B	119.2
C8A—C9A—H9A	119.2	C8B—C9B—H9B	119.2
C9A—C10A—C11A	117.71 (18)	C9B—C10B—C11B	118.18 (17)
C9A—C10A—H10A	121.1	C9B—C10B—H10B	120.9
C11A—C10A—H10A	121.1	C11B—C10B—H10B	120.9
C12A—C11A—C10A	119.24 (17)	C12B—C11B—C10B	118.67 (17)
C12A—C11A—C5A	105.80 (15)	C12B—C11B—C5B	105.53 (15)
C10A—C11A—C5A	134.94 (18)	C10B—C11B—C5B	135.79 (17)
C11A—C12A—O1A	111.54 (15)	C7B—C12B—O1B	123.42 (15)
C11A—C12A—C7A	124.97 (16)	C7B—C12B—C11B	124.93 (16)
O1A—C12A—C7A	123.46 (16)	O1B—C12B—C11B	111.65 (15)
C26A—C21A—C22A	117.97 (17)	C22B—C21B—C26B	117.60 (18)
C26A—C21A—P1A	116.93 (15)	C22B—C21B—P1B	125.56 (14)
C22A—C21A—P1A	124.91 (14)	C26B—C21B—P1B	116.79 (15)
C23A—C22A—C21A	120.67 (18)	C23B—C22B—C21B	121.45 (18)
C23A—C22A—H22A	119.7	C23B—C22B—H22B	119.3
C21A—C22A—H22A	119.7	C21B—C22B—H22B	119.3
C24A—C23A—C22A	120.3 (2)	C24B—C23B—C22B	119.9 (2)
C24A—C23A—H23A	119.9	C24B—C23B—H23B	120.1
C22A—C23A—H23A	119.9	C22B—C23B—H23B	120.1
C23A—C24A—C25A	120.18 (18)	C25B—C24B—C23B	119.5 (2)
C23A—C24A—H24A	119.9	C25B—C24B—H24B	120.2
C25A—C24A—H24A	119.9	C23B—C24B—H24B	120.2
C24A—C25A—C26A	119.49 (19)	C24B—C25B—C26B	120.6 (2)
C24A—C25A—H25A	120.3	C24B—C25B—H25B	119.7
C26A—C25A—H25A	120.3	C26B—C25B—H25B	119.7
C25A—C26A—C21A	121.4 (2)	C25B—C26B—C21B	121.0 (2)
C25A—C26A—H26A	119.3	C25B—C26B—H26B	119.5
C21A—C26A—H26A	119.3	C21B—C26B—H26B	119.5
C32A—C31A—C36A	117.80 (16)	C36B—C31B—C32B	118.20 (18)
C32A—C31A—P1A	124.71 (13)	C36B—C31B—P1B	117.16 (14)
C36A—C31A—P1A	117.15 (13)	C32B—C31B—P1B	124.64 (15)
C33A—C32A—C31A	120.80 (16)	C33B—C32B—C31B	120.61 (19)
C33A—C32A—H32A	119.6	C33B—C32B—H32B	119.7
C31A—C32A—H32A	119.6	C31B—C32B—H32B	119.7
C34A—C33A—C32A	120.72 (17)	C34B—C33B—C32B	120.53 (19)
C34A—C33A—H33A	119.6	C34B—C33B—H33B	119.7
C32A—C33A—H33A	119.6	C32B—C33B—H33B	119.7
C33A—C34A—C35A	119.33 (18)	C33B—C34B—C35B	119.61 (19)
C33A—C34A—H34A	120.3	C33B—C34B—H34B	120.2
C35A—C34A—H34A	120.3	C35B—C34B—H34B	120.2
C36A—C35A—C34A	120.18 (17)	C34B—C35B—C36B	120.05 (19)
C36A—C35A—H35A	119.9	C34B—C35B—H35B	120.0
C34A—C35A—H35A	119.9	C36B—C35B—H35B	120.0
C35A—C36A—C31A	121.15 (17)	C35B—C36B—C31B	120.99 (18)
C35A—C36A—H36A	119.4	C35B—C36B—H36B	119.5
C31A—C36A—H36A	119.4	C31B—C36B—H36B	119.5
C42A—C41A—C46A	117.76 (18)	C46B—C41B—C42B	118.34 (16)
C42A—C41A—P2A	117.35 (14)	C46B—C41B—P2B	125.19 (13)
C46A—C41A—P2A	124.89 (14)	C42B—C41B—P2B	116.47 (13)
C43A—C42A—C41A	121.18 (19)	C43B—C42B—C41B	120.97 (17)
C43A—C42A—H42A	119.4	C43B—C42B—H42B	119.5
C41A—C42A—H42A	119.4	C41B—C42B—H42B	119.5
C44A—C43A—C42A	120.2 (2)	C44B—C43B—C42B	119.72 (17)
C44A—C43A—H43A	119.9	C44B—C43B—H43B	120.1
C42A—C43A—H43A	119.9	C42B—C43B—H43B	120.1
C43A—C44A—C45A	119.7 (2)	C43B—C44B—C45B	120.16 (17)
C43A—C44A—H44A	120.2	C43B—C44B—H44B	119.9
C45A—C44A—H44A	120.2	C45B—C44B—H44B	119.9
C44A—C45A—C46A	120.2 (2)	C44B—C45B—C46B	120.07 (18)
C44A—C45A—H45A	119.9	C44B—C45B—H45B	120.0
C46A—C45A—H45A	119.9	C46B—C45B—H45B	120.0
C45A—C46A—C41A	120.89 (19)	C45B—C46B—C41B	120.73 (17)
C45A—C46A—H46A	119.6	C45B—C46B—H46B	119.6
C41A—C46A—H46A	119.6	C41B—C46B—H46B	119.6
C56A—C51A—C52A	118.26 (16)	C56B—C51B—C52B	118.53 (17)
C56A—C51A—P2A	117.54 (14)	C56B—C51B—P2B	117.23 (15)
C52A—C51A—P2A	124.19 (13)	C52B—C51B—P2B	123.69 (14)
C53A—C52A—C51A	121.12 (17)	C51B—C52B—C53B	120.2 (2)
C53A—C52A—H52A	119.4	C51B—C52B—H52B	119.9
C51A—C52A—H52A	119.4	C53B—C52B—H52B	119.9
C54A—C53A—C52A	119.89 (19)	C54B—C53B—C52B	120.5 (2)
C54A—C53A—H53A	120.1	C54B—C53B—H53B	119.7
C52A—C53A—H53A	120.1	C52B—C53B—H53B	119.7
C53A—C54A—C55A	119.98 (17)	C53B—C54B—C55B	119.83 (18)
C53A—C54A—H54A	120.0	C53B—C54B—H54B	120.1
C55A—C54A—H54A	120.0	C55B—C54B—H54B	120.1
C54A—C55A—C56A	120.15 (18)	C54B—C55B—C56B	119.9 (2)
C54A—C55A—H55A	119.9	C54B—C55B—H55B	120.0
C56A—C55A—H55A	119.9	C56B—C55B—H55B	120.0
C51A—C56A—C55A	120.58 (19)	C55B—C56B—C51B	121.0 (2)
C51A—C56A—H56A	119.7	C55B—C56B—H56B	119.5
C55A—C56A—H56A	119.7	C51B—C56B—H56B	119.5
C6A—O1A—C12A	105.30 (13)	C12B—O1B—C6B	105.27 (13)
C1A—P1A—C21A	103.15 (8)	C21B—P1B—C1B	102.18 (8)
C1A—P1A—C31A	102.08 (8)	C21B—P1B—C31B	101.54 (8)
C21A—P1A—C31A	103.78 (8)	C1B—P1B—C31B	100.47 (8)
C41A—P2A—C51A	102.14 (8)	C7B—P2B—C41B	101.18 (8)
C41A—P2A—C7A	100.61 (8)	C7B—P2B—C51B	103.45 (8)
C51A—P2A—C7A	101.40 (8)	C41B—P2B—C51B	103.28 (8)
			
C6A—C1A—C2A—C3A	−0.4 (3)	C6B—C1B—C2B—C3B	−0.2 (3)
P1A—C1A—C2A—C3A	−176.48 (14)	P1B—C1B—C2B—C3B	−176.01 (14)
C1A—C2A—C3A—C4A	0.1 (3)	C1B—C2B—C3B—C4B	−0.9 (3)
C2A—C3A—C4A—C5A	0.2 (3)	C2B—C3B—C4B—C5B	0.7 (3)
C3A—C4A—C5A—C6A	−0.1 (3)	C3B—C4B—C5B—C6B	0.4 (3)
C3A—C4A—C5A—C11A	−178.70 (19)	C3B—C4B—C5B—C11B	−178.09 (19)
C2A—C1A—C6A—O1A	178.80 (15)	C4B—C5B—C6B—O1B	−179.74 (15)
P1A—C1A—C6A—O1A	−4.9 (2)	C11B—C5B—C6B—O1B	−0.82 (19)
C2A—C1A—C6A—C5A	0.5 (3)	C4B—C5B—C6B—C1B	−1.6 (3)
P1A—C1A—C6A—C5A	176.79 (13)	C11B—C5B—C6B—C1B	177.31 (16)
C4A—C5A—C6A—O1A	−178.72 (15)	C2B—C1B—C6B—C5B	1.4 (3)
C11A—C5A—C6A—O1A	0.23 (19)	P1B—C1B—C6B—C5B	177.55 (13)
C4A—C5A—C6A—C1A	−0.2 (3)	C2B—C1B—C6B—O1B	179.36 (15)
C11A—C5A—C6A—C1A	178.70 (16)	P1B—C1B—C6B—O1B	−4.5 (2)
C12A—C7A—C8A—C9A	−0.4 (3)	C12B—C7B—C8B—C9B	0.3 (2)
P2A—C7A—C8A—C9A	175.70 (14)	P2B—C7B—C8B—C9B	174.34 (14)
C7A—C8A—C9A—C10A	1.4 (3)	C7B—C8B—C9B—C10B	−0.4 (3)
C8A—C9A—C10A—C11A	−1.1 (3)	C8B—C9B—C10B—C11B	0.1 (3)
C9A—C10A—C11A—C12A	−0.1 (3)	C9B—C10B—C11B—C12B	0.3 (3)
C9A—C10A—C11A—C5A	177.58 (19)	C9B—C10B—C11B—C5B	−179.99 (19)
C6A—C5A—C11A—C12A	−0.64 (18)	C6B—C5B—C11B—C12B	0.66 (19)
C4A—C5A—C11A—C12A	178.05 (19)	C4B—C5B—C11B—C12B	179.32 (19)
C6A—C5A—C11A—C10A	−178.57 (19)	C6B—C5B—C11B—C10B	−179.04 (19)
C4A—C5A—C11A—C10A	0.1 (4)	C4B—C5B—C11B—C10B	−0.4 (4)
C10A—C11A—C12A—O1A	179.17 (15)	C8B—C7B—C12B—O1B	−179.80 (15)
C5A—C11A—C12A—O1A	0.86 (19)	P2B—C7B—C12B—O1B	5.8 (2)
C10A—C11A—C12A—C7A	1.3 (3)	C8B—C7B—C12B—C11B	0.1 (3)
C5A—C11A—C12A—C7A	−177.07 (16)	P2B—C7B—C12B—C11B	−174.35 (14)
C8A—C7A—C12A—C11A	−1.0 (3)	C10B—C11B—C12B—C7B	−0.4 (3)
P2A—C7A—C12A—C11A	−177.23 (13)	C5B—C11B—C12B—C7B	179.80 (16)
C8A—C7A—C12A—O1A	−178.66 (15)	C10B—C11B—C12B—O1B	179.47 (15)
P2A—C7A—C12A—O1A	5.1 (2)	C5B—C11B—C12B—O1B	−0.30 (19)
C26A—C21A—C22A—C23A	0.9 (3)	C26B—C21B—C22B—C23B	−1.1 (3)
P1A—C21A—C22A—C23A	−173.89 (15)	P1B—C21B—C22B—C23B	176.26 (15)
C21A—C22A—C23A—C24A	−1.2 (3)	C21B—C22B—C23B—C24B	0.7 (3)
C22A—C23A—C24A—C25A	0.3 (3)	C22B—C23B—C24B—C25B	−0.2 (3)
C23A—C24A—C25A—C26A	0.8 (3)	C23B—C24B—C25B—C26B	0.1 (3)
C24A—C25A—C26A—C21A	−1.1 (3)	C24B—C25B—C26B—C21B	−0.5 (3)
C22A—C21A—C26A—C25A	0.3 (3)	C22B—C21B—C26B—C25B	1.0 (3)
P1A—C21A—C26A—C25A	175.45 (15)	P1B—C21B—C26B—C25B	−176.59 (15)
C36A—C31A—C32A—C33A	1.1 (3)	C36B—C31B—C32B—C33B	−0.9 (3)
P1A—C31A—C32A—C33A	−171.98 (15)	P1B—C31B—C32B—C33B	178.46 (16)
C31A—C32A—C33A—C34A	−1.1 (3)	C31B—C32B—C33B—C34B	−0.4 (3)
C32A—C33A—C34A—C35A	−0.2 (3)	C32B—C33B—C34B—C35B	1.0 (3)
C33A—C34A—C35A—C36A	1.3 (3)	C33B—C34B—C35B—C36B	−0.2 (3)
C34A—C35A—C36A—C31A	−1.3 (3)	C34B—C35B—C36B—C31B	−1.1 (3)
C32A—C31A—C36A—C35A	0.0 (3)	C32B—C31B—C36B—C35B	1.6 (3)
P1A—C31A—C36A—C35A	173.67 (16)	P1B—C31B—C36B—C35B	−177.78 (14)
C46A—C41A—C42A—C43A	−1.0 (3)	C46B—C41B—C42B—C43B	−1.7 (3)
P2A—C41A—C42A—C43A	178.54 (15)	P2B—C41B—C42B—C43B	178.82 (14)
C41A—C42A—C43A—C44A	0.8 (3)	C41B—C42B—C43B—C44B	1.0 (3)
C42A—C43A—C44A—C45A	0.0 (3)	C42B—C43B—C44B—C45B	0.3 (3)
C43A—C44A—C45A—C46A	−0.4 (3)	C43B—C44B—C45B—C46B	−0.9 (3)
C44A—C45A—C46A—C41A	0.0 (3)	C44B—C45B—C46B—C41B	0.1 (3)
C42A—C41A—C46A—C45A	0.6 (3)	C42B—C41B—C46B—C45B	1.2 (3)
P2A—C41A—C46A—C45A	−178.90 (16)	P2B—C41B—C46B—C45B	−179.43 (14)
C56A—C51A—C52A—C53A	0.1 (3)	C56B—C51B—C52B—C53B	−0.8 (3)
P2A—C51A—C52A—C53A	−178.74 (14)	P2B—C51B—C52B—C53B	170.45 (15)
C51A—C52A—C53A—C54A	0.3 (3)	C51B—C52B—C53B—C54B	0.5 (3)
C52A—C53A—C54A—C55A	0.0 (3)	C52B—C53B—C54B—C55B	0.5 (3)
C53A—C54A—C55A—C56A	−0.7 (3)	C53B—C54B—C55B—C56B	−1.2 (3)
C52A—C51A—C56A—C55A	−0.7 (3)	C54B—C55B—C56B—C51B	0.9 (3)
P2A—C51A—C56A—C55A	178.18 (16)	C52B—C51B—C56B—C55B	0.1 (3)
C54A—C55A—C56A—C51A	1.0 (3)	P2B—C51B—C56B—C55B	−171.70 (14)
C1A—C6A—O1A—C12A	−178.20 (16)	C7B—C12B—O1B—C6B	179.72 (15)
C5A—C6A—O1A—C12A	0.28 (18)	C11B—C12B—O1B—C6B	−0.19 (18)
C11A—C12A—O1A—C6A	−0.72 (18)	C5B—C6B—O1B—C12B	0.64 (18)
C7A—C12A—O1A—C6A	177.24 (16)	C1B—C6B—O1B—C12B	−177.53 (16)
C6A—C1A—P1A—C21A	85.02 (15)	C22B—C21B—P1B—C1B	16.60 (18)
C2A—C1A—P1A—C21A	−99.11 (16)	C26B—C21B—P1B—C1B	−166.02 (14)
C6A—C1A—P1A—C31A	−167.50 (14)	C22B—C21B—P1B—C31B	−86.92 (17)
C2A—C1A—P1A—C31A	8.36 (17)	C26B—C21B—P1B—C31B	90.45 (15)
C26A—C21A—P1A—C1A	−175.24 (14)	C6B—C1B—P1B—C21B	75.77 (15)
C22A—C21A—P1A—C1A	−0.44 (18)	C2B—C1B—P1B—C21B	−108.63 (16)
C26A—C21A—P1A—C31A	78.58 (16)	C6B—C1B—P1B—C31B	−179.86 (14)
C22A—C21A—P1A—C31A	−106.62 (17)	C2B—C1B—P1B—C31B	−4.27 (18)
C32A—C31A—P1A—C1A	−112.56 (16)	C36B—C31B—P1B—C21B	−160.73 (15)
C36A—C31A—P1A—C1A	74.27 (16)	C32B—C31B—P1B—C21B	19.90 (19)
C32A—C31A—P1A—C21A	−5.57 (18)	C36B—C31B—P1B—C1B	94.39 (15)
C36A—C31A—P1A—C21A	−178.75 (14)	C32B—C31B—P1B—C1B	−84.98 (18)
C42A—C41A—P2A—C51A	158.45 (14)	C12B—C7B—P2B—C41B	169.08 (13)
C46A—C41A—P2A—C51A	−22.01 (19)	C8B—C7B—P2B—C41B	−4.67 (17)
C42A—C41A—P2A—C7A	−97.31 (15)	C12B—C7B—P2B—C51B	−84.18 (14)
C46A—C41A—P2A—C7A	82.23 (18)	C8B—C7B—P2B—C51B	102.07 (16)
C56A—C51A—P2A—C41A	−92.30 (16)	C46B—C41B—P2B—C7B	81.32 (16)
C52A—C51A—P2A—C41A	86.51 (17)	C42B—C41B—P2B—C7B	−99.27 (14)
C56A—C51A—P2A—C7A	164.08 (15)	C46B—C41B—P2B—C51B	−25.55 (17)
C52A—C51A—P2A—C7A	−17.11 (18)	C42B—C41B—P2B—C51B	153.86 (13)
C8A—C7A—P2A—C41A	8.64 (17)	C56B—C51B—P2B—C7B	−166.96 (14)
C12A—C7A—P2A—C41A	−175.55 (14)	C52B—C51B—P2B—C7B	21.73 (18)
C8A—C7A—P2A—C51A	113.48 (16)	C56B—C51B—P2B—C41B	−61.82 (16)
C12A—C7A—P2A—C51A	−70.72 (15)	C52B—C51B—P2B—C41B	126.87 (16)
